# Genomes are covered with ubiquitous 11 bp periodic patterns, the "class A flexible patterns"

**DOI:** 10.1186/1471-2105-6-206

**Published:** 2005-08-24

**Authors:** Etienne Larsabal, Antoine Danchin

**Affiliations:** 1 Unité de Génétique des Génomes Bactériens, Institut Pasteur, URA CNRS 2171, 28, rue du Docteur Roux, 75724 Paris Cedex 15, France

## Abstract

**Background:**

The genomes of prokaryotes and lower eukaryotes display a very strong 11 bp periodic bias in the distribution of their nucleotides. This bias is present throughout a given genome, both in coding and non-coding sequences. Until now this bias remained of unknown origin.

**Results:**

Using a technique for analysis of auto-correlations based on linear projection, we identified the sequences responsible for the bias. Prokaryotic and lower eukaryotic genomes are covered with ubiquitous patterns that we termed "class A flexible patterns". Each pattern is composed of up to ten conserved nucleotides or dinucleotides distributed into a discontinuous motif. Each occurrence spans a region up to 50 bp in length. They belong to what we named the "flexible pattern" type, in that there is some limited fluctuation in the distances between the nucleotides composing each occurrence of a given pattern. When taken together, these patterns cover up to half of the genome in the majority of prokaryotes. They generate the previously recognized 11 bp periodic bias.

**Conclusion:**

Judging from the structure of the patterns, we suggest that they may define a dense network of protein interaction sites in chromosomes.

## Background

The distribution of nucleotides in genomes is not random, various biases are affecting the genome sequences from organisms spanning the three domains of life. For example, the G+C content affects the genome as a whole.

To visualize the biases in the nucleotides distribution in genomes, investigators have performed a variety of statistical analyses; these operations basically consisted in counting the nucleotides in a variety of subtle ways, while attempting to identify how the counting observed in real examples differed from a random distribution. Relevant statistical methods developed so far include the following: computation of correlations [1], power spectrum analysis [2,3], DNA walking analysis [4], computation of entropy [5,6], Hurst index estimation [7], detrended fluctuation analysis [8], wavelet analysis [9], mutual information function analysis [10], computational linguistics analysis [11].

Among the different biases observed in the nucleotides distribution in genomes, two stood out prominently. Both are short-range biases, i.e. correlating nucleotides over a short distance only, inferior to one thousand base pairs (bp), and both are affecting the genome as a whole. Both are present in many different organisms. This prevalent intensity and ubiquity is a hint that these biases are very likely to be the result of some strong physical constraints and/or biological functions acting on the affected genomes.

The first prevalent bias, the most intense one, is easily visualized in the genomes of all prokaryotes, as well as of lower eukaryotes. It also appears, though very dimly, in the genomes of higher eukaryotes. This bias is periodic with a periodicity of 3 bp (locally, the probability of presence of a given nucleotide depends on its position modulo three). This ubiquitous bias is effectively uncovered by power spectrum analysis [12-17]. Its presence has never been a mystery: it is due to the presence of protein coding genes in genomes. Indeed, the structure of the genetic code strongly affects the distribution of nucleotides within protein coding sequences, biasing the distribution of nucleotide triplets. As the gene density of higher eukaryotes is very small, this bias cannot easily be detected in these organisms. In contrast, for prokaryotes and for lower eukaryotes, in which the gene density is high, this bias is very easily detected. Its association to protein coding proved to be useful to locate exons in higher eukaryotic genomes [18]. This first bias is therefore generated by genomic sequences that are of strong biological significance.

**Figure 1:**
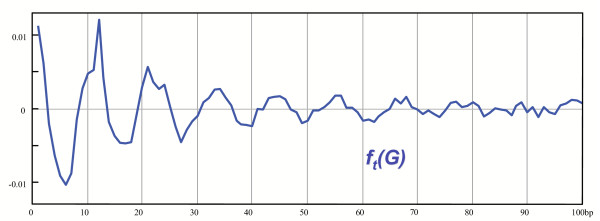
Deconvoluted correlation function of A following A in the genome of *H. pylori*. The correlation function has been treated so as to hide the most intense component of period 3 bp due to the presence of genes in the genome of *H. pylori*. After treatment, the function reveals a prevalent short-range component of period 11 bp. This component represents the prevalent short-range bias of period 11 bp in the distribution of nucleotides in the genome of *H. pylori*.

Likewise, the second prevalent bias, also very intense, is visualized in the genomes of most prokaryotes and lower eukaryotes. For a given genome, the bias is encountered throughout the genome. In contrast with the previous 3 bp periodic bias, which spans large distances (typically several hundreds nucleotides) this bias does not involve nucleotides over a distance longer than about one hundred base-pairs: it is a short-range bias. It is also periodic, but this time with a fuzzy periodicity of mean value 11 bp. This signal has been visualized with the straightforward computation of correlations [1,19] or its equivalent, the power spectrum method [17]. The mean value of the periodicity of this bias varies from organism to organism. In the two articles just mentioned, the authors discuss the relation between phylogeny and the distribution of these periods. It turns out that it is generally of 10 bp for Archaea or hyperthermophilic Bacteria and 11 bp or more for the non-hyperthermophilic Bacteria, though there are many exceptions to this rule [19]. In the case of lower eukaryotes, a period of 10 bp for *C. elegans *and of 11 bp for *S. cerevisiae *has been observed. In the case of higher eukaryotes, a weak bias of period 10 bp is observed once the many repeated sequences present in these genomes have been removed from the analysis [19]. Moreover, in prokaryotes and lower eukaryotes, the bias is affecting coding sequences as well as non-coding sequences. This general observation is illustrated in Figure 1 with a graphic representation of the correlation function of nucleotide A following itself in the genome of *Helicobacter pylori*.

This function measures the probability to get a nucleotide A following another nucleotide A as their distance increases. The correlation function has first been treated by deconvolution so as to hide the overwhelming component of period 3 bp that results from the presence of genes in the genome (see above). The corresponding statistical treatment is described in the Methods section. In the graphic representation of the correlation function shown in Figure 1, there is a prominent component of period 11 bp. It appears as a short-range component as it completely vanishes for nucleotides located more than 70 bp apart. The periodic peaks do not occur every 11 bp exactly but every 10 bp to 12 bp. The strength of the periodic bias is illustrated by their large amplitude.

Although this bias is half as high in intensity as the one created by the presence of genes, and although it is ubiquitous in prokaryotes and lower eukaryotes, the nucleotide sequences generating this bias have not been determined so far. Nonetheless, the biological function that might be at the root of this bias has been proposed. In the case of Archaea, it has been suggested that the positioning of nucleosomes is controlled by some specific sequences, whose nature could however not be identified [1,19].

In the present article, we describe the program we designed, meant to discover the sequences that are generating every short-range bias (excluding the trivial one of period 3 bp generated by the genes) in genomes. Making use of this program, we discovered explicitly the sequences responsible for the bias of period 10–11 bp in the prokaryotic and lower eukaryotic genomes. These sequences, that we named "class A flexible patterns" for reasons that will be clarified in the course of this article, display a new type of organization. We show that the class A flexible patterns are ubiquitous in prokaryotes.

## Results

Our aim was to identify the sequences that generate the 11 bp periodic short-range bias. To address this question, we designed a generic program to determine the sequences that generate any short-range bias in genomes nucleotides distribution (see the Methods section): the sequences responsible for the 11 bp periodic bias should belong to the sequences identified by the program.

For each genome of interest, the output of the program is given as a family of patterns. By pattern, we mean any succession of nucleotides with gaps in between (see the Methods section). The family of patterns returned by the program has the following property: the occurrences in the genome of all the patterns belonging to the pattern family match the sequences of the genome supposed to generate its short-range biases (see Methods section). Because of computation time limitations, our program gives an approximate result only: the patterns shape is restricted and the matching may not be exact (see the Methods and Discussion sections).

The program was run with 49 prokaryotic genomes, with four lower eukaryotic genomes and three viruses sequences. We collected the patterns of all the resulting family of patterns and saw that we could class them into two category of patterns. Naming them after their particular structural features, we called them the "rigid patterns" and the "flexible patterns". The rigid patterns are described first, but not discussed in details because they overlap with previously identified repeated sequences. Then we describe the more frequent but elusive flexible patterns. Among those, a great number belongs to a class that we called the "class A flexible patterns", for reasons explained below. The latter patterns are discussed extensively. Finally, we show that the occurrences of the class A flexible patterns define the sequences generating the bias of period 11 bp in genomes.

### Rigid patterns

A rigid pattern is a pattern verifying the two following properties: first, the distance between the nucleotides making the pattern is the same for every occurrence of the pattern in the genome. Second, some variability in the nature of the nucleotides composing the pattern is allowed from one occurrence to another one. Most patterns described so far in the literature are rigid patterns. For rigid patterns, the exact distances between the nucleotides and the frequency of occurrence of the nucleotides A,T,G,C composing the pattern account for what is usually termed a "consensus sequence".

As a proof of concept, the program uncovered families of rigid patterns in a few selected genomes. Each family was made of short highly repeated motifs. As could be expected, when present in a genome, highly repeated sequences generate a short-range statistical bias. For example, we found the following rigid pattern in the genome of *Escherichia coli *(an x represents any nucleotide):

5**GC**xxx**AT**xxx**GC**xxxxxx**GC**xxx**AT**xxx**GC**-3'

One can recognize in this pattern a consensus for the repeated Bacterial Interspersed Mosaic Elements (BIMEs) sequences of *E. coli *[20]. It is important to note here that, although these sequences are recognized by our program because they create small but significant biases in the nucleotides distribution of *E. coli*, they do not contribute to the generation of the bias of period 11 bp. However, the very fact that we uncovered them is an independent validation of our approach.

### Flexible patterns

To extend the rigid patterns description, we defined the "flexible patterns". A flexible pattern satisfies the two following properties: first, the nature of the nucleotides composing the pattern is the same for all the occurrences of the pattern in a given genome. Second, the distance between the nucleotides composing the pattern varies in a narrow range between occurrences of the pattern. Hence, a flexible pattern differs from a rigid pattern in that it could not generate a "consensus" by aligning sequences without introducing gaps. As an example, here are different occurrences of a flexible pattern found in the genome of

#### Pyrococcus furiosus

**G**xx**A**xxx**TT**xxx**G**xxx**T**

**G**xx**A**xxx**TT**xxx**G**xxx**T**

**GA**xxx**TT**xxxxx**G**xxx**T**

**G**xx**A**xxx**TT**xxx**G**xxxxxx**T**

**G**xxx**A**xxx**TT**xxx**G**xxxxxx**T**

**G**x**A**xxx**TT**xxxx**G**xxxxxx**T**

5'-xxxxxx-20xxxxxxx--3'

From now on, we will represent a given flexible pattern not by its various spellings but by an average representative, in which the distance between the nucleotides is the mean distance of all the distance observed in all the various spellings. For example, we represent the previous flexible pattern by this average representative:

5'-**G**xx**A**xxx**TT**xxx**G**xxxxx**T**-3'

Conversely, in the following, a flexible pattern mentioned by an average representative is defined by the list of similar patterns which are deviating from the average representative by distances varying withing a narrow range between its conserved nucleotides.

The great majority of the patterns that we found by running our program in various genomes turned out to be of the flexible patterns category. We found on average approximately twenty flexible patterns in each genome, be it of a prokaryotic organism or of a lower eukaryotic organism. We observed that the distances between nucleotides composing the flexible patterns we identified vary generally from one to two base pairs. These patterns are composed of five to ten nucleotides spanning a distance of 10 bp to 60 bp. The nucleotides composing these patterns are most of the time either isolated or grouped as dinucleotides.

The description of patterns is limited by our program due to computing time limitations (see the Methods section), for example they cannot be composed of more than six nucleotides. The patterns that we get often seem to be subsets of longer patterns. In the following we mention the longest pattern that can be inferred, but it should be kept in mind that each of its detected variations are composed of only six nucleotides. For example, the following flexible pattern found in *H. pylori*:

5'-**T**xx**A**x**GC**x**TTT**-3'

is defined by the following variations:

**T**xxx**GC**xx**TT**

**T**xxxx**GC**xx**TT**x**T**

**T**xxxx**GC**xx**T**x**TT**

**T**xxxxx**GC**xx**TTT**

**T**xxxxx**A**x**GC**x**TT**

**A**xx**GC**x**TTT**

**A**x**GC**x**TT**x**T**

**A**xx**GC**x**TT**x**T**

### Class A flexible patterns

Among flexible patterns, we observed that a great majority shared a similar structure and were thus easily identifiable. We named "class A flexible patterns" this subset of flexible patterns. We will restrict our study to these patterns, as they account for most, if not all, of the 11 bp period found in the genomes we analyzed.

**Figure 2:**
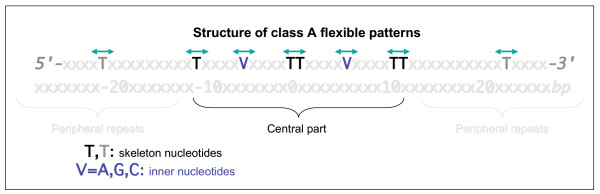
Diagrammatic structure of class A flexible patterns. Class A flexible patterns belong to the category of flexible patterns. Here "flexible" means that there is limited variation in the exact position of their conserved nucleotides. This is shown in the figure by the green arrows, indicating that the position of the conserved nucleotides may vary from one occurrence of the pattern to the next. The particular class of flexible patterns depicted here in the standard 5'-3' orientation is composed of two sets of conserved nucleotides. First, the patterns are shaped by a skeleton of regularly repeated Ts or TTs every 10 bp to 11.5 bp, spanning a maximum of 50 bp. These are called "skeleton nucleotides" and are symbolized by the black and dark grey Ts. The peripheral repeats of the skeleton, in dark grey, are sometimes absent from a given occurrence. The Ts of the central part, spanning 20 bp on average, are always present. Furthermore, class A flexible patterns are composed of a set of "inner nucleotides". These conserved nucleotides are represented here in dark blue. They can be any nucleotide but never Ts. They are located between the Ts of the skeleton and in the central part only.

All class A flexible patterns, though different in spelling, share the same structure, as depicted in Figure 2. The structural features illustrated in this figure are formally defining the class A flexible patterns. The patterns are described here in the standard 5'-3' orientation.

Class A flexible patterns are in total composed of five to ten conserved nucleotides spanning a length of approximately 11 bp to 50 bp. The conserved nucleotides are either isolated or grouped as dinucleotides.

That these patterns belong to the category of flexible patterns is illustrated in Figure 2 by the green arrows above the nucleotides composing the patterns (always isolated nucleotides or dinucleotides). The distance between any of the isolated nucleotides or dinucleotides varies by 1 bp to 2 bp from one occurrence of the pattern to the next in a given genome. Class A flexible patterns are composed of two subsets of conserved nucleotides: the skeleton nucleotides and the inner nucleotides.

The skeleton nucleotides consist of two to five repeats of the single nucleotide T or of the dinucleotide TT, regularly spaced every 10 bp to 11 bp on average. The central part (nucleotides represented in black in Figure 2) is made of two to three repeats. These repeated nucleotides appear at every occurrence of a given pattern in a given genome. Outlying repeats (nucleotides in dark grey in Figure 2) may extend the skeleton outside the central part. Those are involving single nucleotides Ts exclusively and are not always present: they do not appear in every occurrence of a given pattern. Typically, one or two such peripheral repeats of the single nucleotide T on each side of the central part of the skeleton exist in a given occurrence of a pattern. Note that for a given pattern, the distance (averaged over all the occurrences of the given pattern in a given genome) between two neighboring isolated conserved nucleotides Ts or dinucleotides TTs of the skeleton ranges from 7 bp to 12 bp. Yet, the average of these distances over the two to five repeats of the skeleton of the given pattern remains inside the interval of 10 bp to 11.5 bp. The skeleton structure, spanning up to 50 bp in total, is basically the same for all class A flexible patterns, for only the distances between the Ts and the choice of single or dinucleotides can fluctuate.

**Figure 3:**
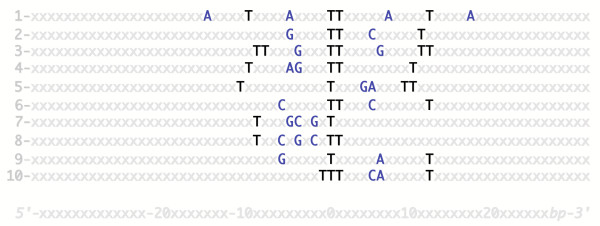
A few identified class A flexible patterns. Ten related yet distinct class A flexible patterns common to different genomes have been identified so far. Their structures share common features, which are characteristic of class A flexible patterns. Peripheral repeats of the skeleton nucleotides of the patterns have not been represented here. Skeleton nucleotides are shown in black. Inner nucleotides are shown in dark blue.

The inner nucleotides consist of one to three conserved nucleotides located exclusively in the central part of the skeleton. Most importantly, these conserved nucleotides are found to be either A, G or C (a particular nucleotide specifying the particular kind of pattern identified, see Figure 3) but never T. They are either isolated or grouped as dinucleotides (isolated conserved nucleotides are more frequent than conserved dinucleotides). There can be only one isolated nucleotide or dinucleotide between two neighboring skeleton nucleotides. The position of the inner nucleotides is usually located exactly in the middle of two neighboring Ts of the skeleton. These inner nucleotides play a discriminating role in class A flexible patterns as they differentiate patterns from one another.

The central part of these patterns is composed of three to six skeleton nucleotides and of two to four inner nucleotides (see Figure 2). Altogether, the central part is composed on average of six conserved nucleotides covering from 10 bp to 33 bp. This part of the patterns is the one that varies from one class A flexible pattern to another, both in the choice of single or dinucleotides in the skeleton and in the nature of the inner nucleotides. Therefore, we choose to subsequently identify the patterns using this central part only.

The program we ran is limited to identification of patterns spanning up to a maximum of 60 bp (see the Methods section). This implies that we may have been missing some peripheral repeats of Ts in some occurrences of the patterns, but we did not miss important nucleotides as the latter are located in the central parts of the patterns only.

### Distribution of class A flexible patterns in organisms

As a whole, cumulating all the tested genomes, we could identify twenty different types of class A flexible patterns. Some genomes harbor specific class A flexible patterns that are found in no other genome. In contrast, some types of patterns are found in more than one genome. We could identify ten such conserved types of patterns. In Figure 3, we list these ten types of class A flexible patterns.

Patterns numbered 1 to 5 in Figure 3 are present in many genomes, patterns numbered 6 to 10 are present in less than ten different genomes.

In Table 1, we display the organisms in which these patterns were identified, as well as the phylogenetic family to which the organisms belong. It turned out that every one of the 49 prokaryotic genomes tested, two of the four lower eukaryotic genomes tested (*Saccharomyces cerevisiae *and *Caenorhabditis elegans*) and the two genomes of bacteriophages analyzed were harboring class A flexible patterns.

**Table 1:** Distribution of class A flexible patterns in genomes.

	**1**	**2**	**3**	**4**	**5**	**6**	**7**	**8**	**9**	**10**	
											
*Aeropyrum pernix*	X		X	X							Archaea; Crenarchaeota; Thermoprotei; Desulfurococcales
*Sulfolobus solfataricus*	X		X	X	X						Archaea; Crenarchaeota; Thermoprotei; Sulfolobales
*Sulfolobus tokodaii*			X	X	X						Archaea; Crenarchaeota; Thermoprotei; Sulfolobales
*Pyrobaculum aerophilum*		X								X	Archaea; Crenarchaeota; Thermoprotei; Thermoproteales
*Archaeoglobus fulgidus*			X	X	X						Archaea; Euryarchaeota; Archaeoglobi; Archaeoglobales
*M. Acetivorans*	X		X	X	X		X	X			Archaea; Euryarchaeota; Methanosarcinales
*Halobacterium sp.*			X								Archaea; Euryarchaeota; Halobacteriales
*M. thermoautotrophicum*			X	X							Archaea; Euryarchaeota; Methanobacteriales
*Methanococcus jannashii*	X			X	X						Archaea; Euryarchaeota; Methanococcales
*Pyrococcus abyssi*			X	X	X						Archaea; Euryarchaeota; Thermococcales
*Pyrococcus furiosus*	X		X	X	X						Archaea; Euryarchaeota; Thermococcales
*Pyrococcus horikoshii*			X	X	X						Archaea; Euryarchaeota; Thermococcales
*Thermoplasma acidophilum*				X		X					Archaea; Euryarchaeota; Thermoplasmatales
*Tropheryma whipplei*		X	X						X		Bacteria; Actinobacteria; Actinomycetales
*Aquifex aeolicus*	X			X					X		Bacteria; Aquificae; Aquificales
*Chlorobium tepidum*	X						X				Bacteria; Chlorobi; Chlorobiales
*Synechocystis sp.*											Bacteria; Cyanobacteria; Chroococcales
*Deinococcus radiodurans*		X						X			Bacteria; Deinococcus-Thermus; Deinococcales
*Bacillus subtilis*	X							X			Bacteria; Firmicutes; Bacillales
*Oceanobacillus iheyensis*	X										Bacteria; Firmicutes; Bacillales
*Listeria monocytogenes*	X					X					Bacteria; Firmicutes; Bacillales
*T. Tengcongensis*	X		X								Bacteria; Firmicutes; Clostridia; Thermoanaerobacteriales
*Streptococcus pneumoniae*	X										Bacteria; Firmicutes; Lactobacillales
*Pirellula sp.*	X										Bacteria; Planctomycetes; Planctomycetales
*Magnetactic cocci*		X								X	Bacteria; Proteobacteria
*Caulobacter vibrioides*		X									Bacteria; Proteobacteria; Alphaproteobacteria; Caulobacteriales
*Agrobacterium tumefaciens*		X						X			Bacteria; Proteobacteria; Alphaproteobacteria; Rhizobiales
*Sinorhizobium meliloti*								X			Bacteria; Proteobacteria; Alphaproteobacteria; Rhizobiales
*Rickettsia conorii*	X		X				X				Bacteria; Proteobacteria; Alphaproteobacteria; Rickettsialles
*Rickettsia prowozekii*	X	X	X		X		X				Bacteria; Proteobacteria; Alphaproteobacteria; Rickettsialles
*Bordetella pertussis*	X						X				Bacteria; Proteobacteria; Betaproteobacteria; Burkholderiales
*Neisseria meningitidis*		X									Bacteria; Proteobacteria; Betaproteobacteria; Neisseriales
*Campylobacter jejuni*	X	X									Bacteria; Proteobacteria; Epsilonproteobacteria; Campylobacterales
*Helicobacter hepaticus*	X	X	X	X							Bacteria; Proteobacteria; Epsilonproteobacteria; Campylobacterales
*Helicobacter pylori*		X	X				X				Bacteria; Proteobacteria; Epsilonproteobacteria; Campylobacterales
*Wolinella succinogenes*		X	X		X						Bacteria; Proteobacteria; Epsilonproteobacteria; Campylobacterales
*P. haloplanktis*		X					X				Bacteria; Proteobacteria; Gammaproteobacteria; Alteromonadales
*Candidatus bl. floridanus*	X										Bacteria; Proteobacteria; Gammaproteobacteria; Enterobacteriales
*Buchnera aphidicola*	X										Bacteria; Proteobacteria; Gammaproteobacteria; Enterobacteriales
*Escherichia coli*		X									Bacteria; Proteobacteria; Gammaproteobacteria; Enterobacteriales
*Wigglesworthia glossinidia*	X										Bacteria; Proteobacteria; Gammaproteobacteria; Enterobacteriales
*Coxiella burnetii*	X										Bacteria; Proteobacteria; Gammaproteobacteria; Legionellales
*Haemophilus influenzae*	X	X			X	X	X				Bacteria; Proteobacteria; Gammaproteobacteria; Pasteurellales
*Pseudomonas aeruginosa*		X						X			Bacteria; Proteobacteria; Gammaproteobacteria; Pseudomonadales
*Pseudomonas putida*	X	X					X	X			Bacteria; Proteobacteria; Gammaproteobacteria; Pseudomonadales
*Vibrio vulnificus*		X				X	X				Bacteria; Proteobacteria; Gammaproteobacteria; Vibrionales
*Xylella fastidiosa*	X	X			X	X		X			Bacteria; Proteobacteria; Gammaproteobacteria; Xanthomonadales
*Leptospira interrogans*	X										Bacteria; Spirochaetes; Spirochaetales
*Thermotoga maritima*	X								X		Bacteria; Thermotogae; Thermotogales
*Plasmodium falciparum*											Eukaryota; Alveolata; Apicomplexa
*Saccharomyces cerevisiae*	X				X						Eukaryota; Fungi; Ascomycota
*Encephalitozoon cuniculi*											Eukaryota; Fungi; Microsporidia
*Caenorhabditis elegans*	X		X		X						Eukaryota; Metazoa; Nematoda
*Enterobacteria phage T4*	X										Virus; Enterobacteria phage T4
*S. tengcon.. Vvrus STSV1*	X										Virus; Fusellovirus
*Human herpesvirus 4*											Virus; Human herpesvirus 4

**1. A**xxxx**T**xxxx**A**xxxx**TT**xxxxx**A**xxxx**T**xxxx**A**

**2. G**xxxx**TT**xxx**C**xxx**T**

**3. TT**xxx**G**xxx**TT**xxxx**G**xxxx**TT**

**4. T**xxxx**AG**xxx**TT**xxxxxxxx**T**

**5. T**xxxxxxxxxx**T**xxx**GA**xxx**TT**

**6. C**xxxxx**TT**xxx**C**xxxxxx**T**

**7. T**xxx**GC**x**G**x**T**

**8. T**xx**C**x**G**x**C**x**TT**

**9. G**xxxxx**T**xxxxx**A**xxxxx**T**

**10. TTT**xxx**CA**xxxxx**T**

First, we found out that class A flexible patterns are ubiquitous in prokaryotes. Indeed, each of 49 genomes of prokaryotes tested harbors one or more different types of class A flexible patterns. The genome of *Xylella fastidiosa *harbors for instance five different types of patterns. Usually, each genome harbors two to four different types of class A flexible patterns. Second, each of the patterns numbered 1 through 5 in Figure 3 is present in more than 10 different genomes. This makes it possible to discuss the nature of the distribution of these five types of patterns in genomes.

Pattern 1 has been detected in more than 50% of the 56 tested genomes, with no relationship to phylogenetic branches as we found it in Archaea, in Bacteria, in lower eukaryotes and in phages (see Table 1). This pattern alone may be ubiquitous as a low content of this pattern in a given genome would fail to be detected by our approach.

Pattern 2 is present in a total of 19 genomes. Out of these 19 genomes, 16 belong to *Proteobacteria*. Three further genomes, that do not belong to the *Proteobacteria *clade, display this type of pattern. Among those, we found first two Bacteria: *Deinococcus radiodurans *and *Tropheryma whipplei*. The former lives under highly desiccated or radiation-exposed conditions, with remarkable features in DNA maintenance [21], while the latter is a highly degenerate parasite [22]. The third organism which is not a *Proteobacteria *and where this type of pattern is present is an Archaeon: *Pyrobaculum aerophilum *[23]. Overall, the distribution of pattern number 2 in genomes is highly correlated with the *Proteobacteria *class of organisms. It is present throughout this class of organisms as it has been detected in some genomes of the alpha, beta, epsilon and gamma groups (the delta group has not yet been analyzed). It is also remarkably present in all tested genomes of the epsilon group.

Pattern 3 is present in 18 genomes in total, in Archaea, in Bacteria and in lower eukaryotes. Pattern 4 is present in 13 genomes in all. It has been identified in 11 of the 13 archaeal genomes analyzed (in *Crenarcheota *as well as in *Euryarchaeota*). It is also present in two Bacteria (*Aquifex aeolicus *and *Helicobacter hepaticus*). Hence, the distribution of this pattern in genomes seems to be somewhat correlated with the archaeal kingdom.

**Figure 4:**
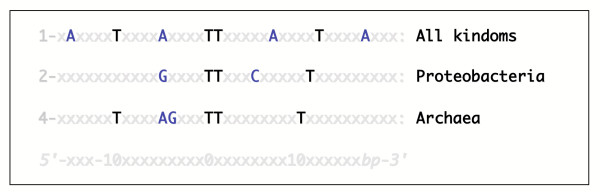
The distribution of three types of class A flexible patterns is correlated to specific phylogenetic groups of organisms. We identified five class A flexible patterns distributed in many different organisms. Three of them, displayed here, show a distribution which can be related to the phylogeny.

Pattern 5 is present in 14 genomes in total, in Archaea, in Bacteria and in lower eukaryotes. The other identified class A flexible patterns are present in only a few organisms. Moreover, these organisms do not clearly belong to any specific phylogenetic lineage. In Figure 4 are summarized the few parallels that could be drawn between the distribution of class A flexible patterns and phylogeny. Each of these three patterns is present in more than 10 genomes out of the 56 tested.

### Distribution of class A flexible patterns in a given genome

The occurrences of class A flexible patterns are equally distributed in the two strands of chromosomes. These occurrences cover a considerable part of each genome. The conserved nucleotides of all occurrences of all class A flexible patterns are involving up to one fourth of the total number of nucleotides of a given genome (24% in the case of *H. pylori*). If we take into consideration the total length that the occurrences of the patterns span in a genome, then it comes up to one half of each genome (51% in the case of *H. pylori*). In the case of *H. pylori*, the span of the patterns ranges from 9 bp to 29 bp (Table 2). We observed that the patterns' occurrences can be overlapping. Interestingly, class A flexible patterns occur indifferently in coding and in non-coding regions of genomes. They are neither correlated with the leading nor with the lagging strand of chromosomes. All things considered, there seems to be no obvious bias in the distribution of the occurrences of the patterns.

**Table 2:** The variations defining the five class A flexible patterns found in the genome of *H. pylori*

1-5'-xxxxxxxxxxxxxxx**T**xxxxxxxxxx**T**xxxx**G**xxx**TT**x**T**xxxxxxxxxxxxxxxxxxxxx-3'
xxxxxxxxxxxxxxxxxxxx**T**xxxxxxxxxx**T**xxxx**G**xxx**TT**x**T**xxxxxxxxxxxxxxxxxxxxxxxx
xxxxxxxxxxxxxxxxxxx**T**xxxxxxxxxxx**T**xxxx**G**xxx**TT**x**T**xxxxxxxxxxxxxxxxxxxxxxxx
xxxxxxxxxxxxxx-20xxxxxxx-10xxxxxxxxx0xxxxxxxxx10xxxxxxxx20xxxxxxxx30

2-5'-xxxxxxxxxxxxxx**GG**xx**TTT**xxxxxxxxxx**T**xxxxxxxxx**T**xxxxxxxxxxxxxxxxxx-3'
xxxxxxxxxxxxxxxxxxx**G**xxx**T**x**TT**xxxxxxxxx**T**xxxxxxxxxx**T**xxxxxxxxxxxxxxxxxxxx
xxxxxxxxxxxxxxxxxxxx**G**xx**TTT**xxxxxxxxx**T**xxxxxxxxxx**T**xxxxxxxxxxxxxxxxxxxxx
xxxxxxxxxxxxxxxxxxxx**G**xx**TTT**xxxxxxxxx**T**xxxxxxxxxxx**T**xxxxxxxxxxxxxxxxxxxx
xxxxxxxxxxxxxxxxxxxx**G**xx**TTT**xxxxxxxxxx**T**xxxxxxxxx**T**xxxxxxxxxxxxxxxxxxxxx
xxxxxxxxxxxxxxxxxxx**GG**xx**TTT**xxxxxxxxxx**T**xxxxxxxxxxxxxxxxxxxxxxxxxxxxxxx
xxxxxxxxxxxxxxxxxxx**GG**xx**TT**x**T**xxxxxxxxx**T**xxxxxxxxxxxxxxxxxxxxxxxxxxxxxxx
xxxxxxxxxxxxxxxxxxxx**G**xx**TTT**xxxxxxxxx**TT**xxxxxxxxxxxxxxxxxxxxxxxxxxxxxxx
xxxxxxxxxxxxxxxxxx**G**x**G**xx**TTT**xxxxxxxxxx**T**xxxxxxxxxxxxxxxxxxxxxxxxxxxxxxx
xxxxxxxxxxxxxx-20xxxxxxx-10xxxxxxxxx0xxxxxxxxx10xxxxxxxx20xxxxxxxx30

3-5'-xxxxxxxxxxxxxxxx**T**xxxxxxxxx**TTT**xx**AA**x**C**xx**T**xxxxxxxxxxxxxxxxxxxxxx-3'
xxxxxxxxxxxxxxxxxxxxx**T**xxxxxxxxxx**TT**xx**A**xx**C**xx**T**xxxxxxxxxxxxxxxxxxxxxxxxx
xxxxxxxxxxxxxxxxxxxxxx**T**xxxxxxxxxx**T**xx**AA**x**C**xx**T**xxxxxxxxxxxxxxxxxxxxxxxxx
xxxxxxxxxxxxxxxxxxxxx**T**xxxxxxxxxxx**T**xx**AA**x**C**xx**T**xxxxxxxxxxxxxxxxxxxxxxxxx
xxxxxxxxxxxxxxxxxxxxxx**T**xxxxxxxxx**TT**xx**AA**xx**C**xxxxxxxxxxxxxxxxxxxxxxxxxxx
xxxxxxxxxxxxxxxxxxxxxxxxxxxxxxxxxx**T**x**AA**x**CC**x**T**xxxxxxxxxxxxxxxxxxxxxxxxx
xxxxxxxxxxxxxxxxxxxxxxxxxxxxxxxxx**T**xx**AA**x**CC**xx**T**xxxxxxxxxxxxxxxxxxxxxxxx
xxxxxxxxxxxxxxxxxxxxxxxxxxxxxxxx**TT**xx**A**x**CC**xx**T**xxxxxxxxxxxxxxxxxxxxxxxxx
xxxxxxxxxxxxxxxxxxxxxxxxxxxxxxx**TTT**xx**A**xx**C**xx**T**xxxxxxxxxxxxxxxxxxxxxxxxx
xxxxxxxxxxxxxxxxxxxxxxxxxxxxx**TT**x**T**xxx**A**xx**C**xx**T**xxxxxxxxxxxxxxxxxxxxxxxxx
xxxxxxxxxxxxxxxxxxxxxxxxxxxxxx**T**x**TT**xx**A**xx**C**xx**T**xxxxxxxxxxxxxxxxxxxxxxxxx
xxxxxxxxxxxxxx-20xxxxxxx-10xxxxxxxxx0xxxxxxxxx10xxxxxxxx20xxxxxxxx30

4-5'-xxxxxxxxxxxxxxxxxxxxxxxxxx**GG**xx**TTT**xxxxx**C**xxxxxxxxxxxxxxxxxxxxx-3'
xxxxxxxxxxxxxxxxxxxxxxxxxxxxxxxx**GG**x**TTT**xxxx**C**xxxxxxxxxxxxxxxxxxxxxxxxx
xxxxxxxxxxxxxxxxxxxxxxxxxxxxxxx**GG**xx**TT**x**T**xxxx**C**xxxxxxxxxxxxxxxxxxxxxxxx
xxxxxxxxxxxxxxxxxxxxxxxxxxxxxxx**GG**xx**T**x**TT**xxxx**C**xxxxxxxxxxxxxxxxxxxxxxxx
xxxxxxxxxxxxxxxxxxxxxxxxxxxxxxx**G**x**G**x**T**x**TT**xxxx**C**xxxxxxxxxxxxxxxxxxxxxxxx
xxxxxxxxxxxxxxxxxxxxxxxxxxxxxx**G**x**G**xx**TTT**xxxx**C**xxxxxxxxxxxxxxxxxxxxxxxxx
xxxxxxxxxxxxxx-20xxxxxxx-10xxxxxxxxx0xxxxxxxxx10xxxxxxxx20xxxxxxxx30

5-5'-xxxxxxxxxxxxxxxxxxxxxxxxxx**T**xx**A**x**GC**x**TTT**xxxxxxxxxxxxxxxxxxxxxxx-3'
xxxxxxxxxxxxxxxxxxxxxxxxxxxxxxxx**T**xxx**GC**xx**TT**xxxxxxxxxxxxxxxxxxxxxxxxxx
xxxxxxxxxxxxxxxxxxxxxxxxxxxxxxx**T**xxxx**GC**xx**TT**x**T**xxxxxxxxxxxxxxxxxxxxxxxx
xxxxxxxxxxxxxxxxxxxxxxxxxxxxxxx**T**xxxx**GC**xx**T**x**TT**xxxxxxxxxxxxxxxxxxxxxxxx
xxxxxxxxxxxxxxxxxxxxxxxxxxxxxx**T**xxxxx**GC**xx**TTT**xxxxxxxxxxxxxxxxxxxxxxxxx
xxxxxxxxxxxxxxxxxxxxxxxxxxxx**T**xxxxx**A**x**GC**x**TT**xxxxxxxxxxxxxxxxxxxxxxxxxxx
xxxxxxxxxxxxxxxxxxxxxxxxxxxxxxxxx**A**xx**GC**x**TTT**xxxxxxxxxxxxxxxxxxxxxxxxxx
xxxxxxxxxxxxxxxxxxxxxxxxxxxxxxxxxx**A**x**GC**x**TT**x**T**xxxxxxxxxxxxxxxxxxxxxxxxx
xxxxxxxxxxxxxxxxxxxxxxxxxxxxxxxxx**A**xx**GC**x**TT**x**T**xxxxxxxxxxxxxxxxxxxxxxxxx
xxxxxxxxxxxxxx-20xxxxxxx-10xxxxxxxxx0xxxxxxxxx10xxxxxxxx20xxxxxxxx30

|p463pt|The variations defining the five class A flexible patterns found in the genome of*H. pylori*

### Contribution of class A flexible patterns to the 11 bp periodic bias

The structure of class A flexible patterns is highly reminiscent of the 11 bp periodic bias in genomes of prokaryotes and lower eukaryotes. Indeed, the patterns have a core of repeated Ts or TTs every 10 bp-11 bp on average in all occurrences. It can therefore be expected that because these periodic nucleotides are densely spread, a bias of period 10 bp-11 bp will be generated in the corresponding genome sequences. The length of the patterns when the peripheral repeats are considered (up to 60 bp) is on the same order as the span of the 10 bp-11 bp periodic component in the correlation between nucleotides (see Figure 1). Furthermore, we systematically observed that the component of period 11 bp is somewhat fuzzy (see the blunt shaped peaks in Figure 1). This is consistent with the fact that the distance between neighboring skeleton nucleotides ranges from 7 bp to 12 bp. This is also consistent with the involvement of dinucleotides in class A flexible patterns. Finally, the occurrences of class A flexible patterns distribute throughout a given genome, with no apparent preference for coding or non-coding regions, similarly to the bias of period 10–11 bp. Now we want to show that the class A flexible patterns are indeed the source of the 11 bp periodic bias in genomes. We illustrate this with the genome of *H. pylori *as the statistical bias of period 11 bp is particularly prominent there. We got the same results for all other genomes analyzed.

The class A flexible patterns discovered in the *H. pylori *genome are the following:

1-5'-**T**xxxxxxxxxx**T**xxxx**G**xxx**TT**x**T**-3'

2-5'-**GG**xx**TTT**xxxxxxxxxx**T**xxxxxxxxx**T**-3'

3-5'-**T**xxxxxxxxx**TTT**xx**AA**x**C**xx**T**-3'

4-5'-**GG**xx**TTT**xxxxx**C**-3'

5-5'-**T**xx**A**x**GC**x**TTT**-3'

Patterns numbered from 1 to 3 are also found in genomes of other organisms, while patterns 4 and 5 are found only in this genome. *Helicobacter pylori *is remarkable as the skeleton nucleotides are composed of the trinucleotide TTT. For each of those flexible patterns, Table 2 illustrates the list of their variations. No peripheral repeats are displayed, as we failed to determine any in this particular genome. It is interesting to note that all the variations of these five patterns are indeed over-represented in the genome of *H. pylori*. We compared the number of occurrences of the patterns in the authentic genome to the number of occurrences in a model genome that keeps only the crude statistical features of the nucleotide distribution in the *H. pylori *genome (see the Method section). We found that the variations of pattern 1 occur approximately 30% more often in the authentic genome than in the model genome, the variations of pattern 2 approximately 40%, the variations of pattern 3 approximately 30%, the variations of pattern 4 approximately 40%, the variations of pattern 5 approximately 30%. All the nucleotides involved in the occurrences of patterns 1 to 5 and of their reverse complements amount to 24% of the total number of nucleotides contained in the whole genome. To explore whether the bias of period 11 bp in the distribution of the nucleotides is due to these 24% of the genome of *H. pylori*, we constructed two reference genomes for comparison.

**Figure 5:**
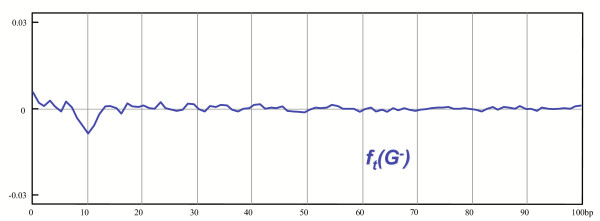
The treated correlation function of *G*_*mo*_(*G*^-^). This correlation function of nucleotide A following A reveals biases generated by the part of the genome of *H. pylori *that do not contain occurrences of class A flexible patterns.

We constructed a first "deconvoluted" genome *G*_*mo*_(*G*^-^) in the following way (see the Methods section): starting from the authentic genome of *H. pylori*, every nucleotide which belongs to any occurrence of any of the five class A flexible patterns or of their reverse complements is replaced by the nucleotide of a model genome preserving the local composition in hexanucleotides of the authentic genome but not their order (see the Methods section) while every other nucleotide is kept unaltered. We plotted the treated correlation function of *G*_*mo*_(*G*^-^) for the nucleotide A following A (see the Methods section) in Figure 5. The 11 bp periodic bias is now absent from this plot. This means that the 76% of the genome of *H. pylori *which is not covered by class A flexible patterns does not have any significant 11 bp periodic statistical bias. Hence, we concluded that class A flexible patterns are generating the 11 bp bias in genomes.

**Figure 6:**
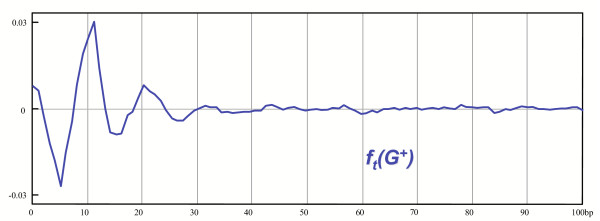
The treated correlation function of *G*_*mo*_(*G*^+^). This correlation function of nucleotide A following A reveals biases in the genome of *H. pylori *which are generated by the occurrences of class A flexible patterns in its genome.

Interestingly, the 11 bp periodic bias disappeared even at correlations over 30 bp, despite the fact that our patterns are never longer that 30 bp for this genome (we have deconvoluted the central parts of the patterns but not the hypothetical peripheral repeats). Deprived of the core sequences of the patterns, the peripheral repeats, even if they exist, can no longer generate much bias. In Figure 5, one can notice a small peak pointing downwards at 11 bp. This probably reflects the fact that we failed to describe accurately the patterns and therefore removed too many sequences, some of which artefactually taken as genuine patterns. Second, we plotted the treated correlation function (see the Methods section) of a complementary model: *G*_*mo*_(*G*^+^), the "convoluted" genome (Figure 6). As in the preceding model, *G*_*mo*_(*G*^+^) is built starting from the authentic genome of *H. pylori*: all the nucleotides not belonging to occurrences of class A flexible patterns and of their reverse complements are replaced by the nucleotides of a model genome (see the Methods section). The 11 bp statistical bias from the original genome is now visible again (the treated correlation function of the original genome is shown in Figure 1). The correlations over 30 bp are hardly visible, which is consistent with the fact that no peripheral repeats were introduced in the convolution process. In this "realistic" imitation of the *H. pylori *genome, the correlations below 30 bp are somewhat too intense when compared to the real ones, displayed in Figure 1. This shows again that we removed too many core sequences, as they were not described with enough accuracy. The sum of the treated correlation function of the deconvoluted genome and of the treated correlation function of the convoluted genome fails to be exactly equal to the treated correlation function of the authentic genome. This shows that there exist correlations between occurrences of class A flexible patterns and of neighboring sequences. It can be expected that these correlations involve the undetected peripheral repeats.

Finally we must note that we chose to illustrate the relationship between class A flexible patterns and the 11 bp bias with the correlation function calculated for an A following an A, as the correlations are specially strong for those two nucleotides. However the results reported are still valid for any combination of two nucleotides.

## Discussion

In the present work we focused on class A flexible patterns as they are the source of the 11 bp periodic bias long known to exist in genomes. Because of the technical limitations of our approach we expect that there may still be other classes of flexible patterns in DNA sequences. They must be however relatively less important as genome sequences do not display prominent short-range biases other than the 3 bp and the 11 bp periodic long identified, while deconvolution of authentic genome sequences from the patterns we identified yielded sequences which no longer displayed any outstanding periodicity.

### Limitations in the description of class A flexible patterns

As explained in the Methods section, our approach suffers some limitations, mainly due to computational time limitations. First, simply for stochastic reasons (the signal must be significantly higher than the noise), we would not find sequences that are generating weak biases or that are present in a too limited amount in genomes (with a frequency below ). Hence we probably missed the presence of some class A flexible patterns in some genomes. Second, the output of our program may have been somewhat inaccurate. Namely, because of the limitation we had to impose on the correlations order (see the Methods section), we may have identifed some patterns as genuine while they would represent a mix of different patterns present at distinct locations in the genomes. Third, we are bound to miss completely any pattern in which the shorter distance between conserved nucleotides is longer than 14 bp (see the Methods section). Fourth, the patterns spellings are but an approximation. Our program has restrictions in the maximum length and number of conserved nucleotides of patterns it is able to determine. As a consequence, we may have missed peripheral parts of the patterns we identified. Still, these restrictions probably did not affect much our spelling of class A flexible patterns, as these patterns are short enough: the central parts span only 20 bp on average. In contrast, in the identification of rigid patterns, typically made of continuous sequences of conserved nucleotides ("words" or "motifs"), we could not retrieve all conserved nucleotides. This was not, however, the main goal of this work.

### Connection to optimal growth temperature

As phylogeny cannot account for the distribution of patterns numbered 3 and 5 in Figure 3, we may wonder whether the distribution of these two class A flexible patterns could be related to physical or biological parameters of the organisms in which they have been identified. We took into account the Gram staining, the cell shape, oxygen dependency, sporulation ability, encapsulation ability, optimal pH and maximum growth temperature, GC content and GC skew. Among those features, the optimal growth temperature somewhat correlates with the distribution of these class A flexible patterns. Indeed, both patterns are present mostly in thermophilic organisms. Still, it remains difficult to draw any firm conclusion in this matter as all tested Archaea but one (*Methanosarcina acetivorans*) are thermophilic and as these patterns are found mostly in Archaea. The question thus arises to determine whether these patterns are present in archaeal organisms or in thermophilic organisms. It is not yet possible to draw a clear rule from the presently tested genomes.

### Class A flexible patterns may define protein interaction sites on the DNA molecule

The very structure of class A flexible patterns offers precious hints to conjecture their biological function. The hypothesis we propose is that the patterns are the signatures of DNA-protein interaction sites. Five arguments tend to support this idea. These are only theoretical arguments and our hypothesis needs to be substantiated by further experiments. First argument: to our knowledge, the length of class A flexible patterns is in a range appropriate for DNA-protein interactions. The total length of the patterns ranges from 11 bp to 60 bp while the length of the central part ranges from 10 bp to 33 bp (see Figure 2). The size of the DNA-protein binding sites usually ranges from 10 bp to 40 bp [24,25]. Hence the central part of the patterns, which is specific and conserved, may be the interacting protein-DNA interface.

Second argument: the number of conserved nucleotides composing the central parts of class A flexible patterns (six on average, see Figure 2) is compatible with the hypothesis. Indeed, if more nucleotides were conserved in the sequence, it is likely that the interaction would be very strong and would therefore have been already identified. Furthermore it would correspond to a stable interaction that would presumably preclude any function of the DNA molecule requiring its opening. In contrast, if there were fewer conserved nucleotides, the interaction would be too weak to create a specific interaction with proteins. Previous studies have established that the average number of conserved nucleotides in DNA-protein interaction sites ranges from five to ten conserved nucleotides [25].

**Figure 7:**
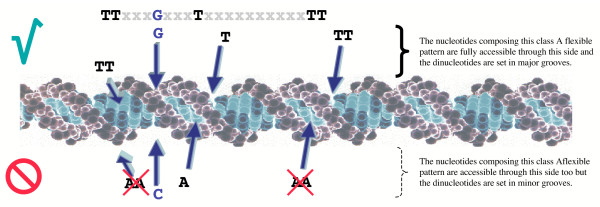
Accessibility of class A flexible patterns. There are two opposed sides from which nucleotides composing this occurrence of this given class A flexible pattern are accessible. The dinucleotides are visible through major grooves only from the upper side and hence fully accessible from this side only. Hence a given occurrence of a given class A flexible pattern in a genome is only accessible from one side of the DNA molecule.

Third argument: the position of the conserved nucleotides of class A flexible patterns is remarkably consistent with the hypothesis of a DNA-protein interaction site. Class A flexible patterns are composed of a skeleton made of regularly repeated Ts or TTs every 10 bp-11.5 bp on average. As the shape of the DNA molecule is helical, with a pitch of average 10.5 bp, varying from 10 bp to 12 bp [26], when unbound, repeated conserved nucleotides of the skeleton always appear at the same side of the helix, in the major groove and in the minor groove respectively (see Figure 7). Inner nucleotides of the patterns, which are always A, G or C depending on the particular pattern considered, are set between the repeated Ts of the skeleton, most often in the middle of two neighboring repeats. Hence, the inner nucleotides also appear on the same two sides of the DNA molecule, through grooves that are opposite to those of the skeleton nucleotides. Note that interactions between proteins and DNA minor grooves are well documented [27,28].

The spatial structure of the DNA molecule of class A flexible patterns is illustrated in Figure 7. The nucleotides composing the example pattern of the figure are accessible from the upper side, with the skeleton nucleotides visible through major grooves and the inner nucleotides visible through minor grooves, or from the lower side, with the skeleton nucleotides visible through minor grooves and the inner nucleotides visible through major grooves.

The skeleton of the patterns is half composed of repeated dinucleotides TTs. In contrast, inner nucleotides are mostly isolated conserved nucleotides. A dinucleotide may be less easily accessed through a minor groove because this groove is too narrow. Conversely, it may be easily accessed through a major groove as the latter is wider. Hence, class A flexible patterns may be actually accessible by only one of the two opposed sides of the DNA double helix, the one where skeleton nucleotides are seen through major grooves, as shown in Figure 7. This gives a very specific argument to think that the function of these patterns may be to define interaction sites with some proteins. Indeed, a protein interacting with the DNA molecule usually comes along one defined side of the molecule and at any rate is never covering the molecule on all sides [29]. The position of the nucleotides composing the patterns is fully consistent with this requirement.

Fourth argument: class A flexible patterns belong to the group of flexible patterns. This means that the exact position of conserved nucleotides of the patterns varies from one occurrence of the patterns in genomes to the next one. This property is fully consistent with the hypothesis that the patterns are signatures of motifs allowing interaction with a geometrically rigid protein, as explained below.

The DNA molecule is a flexible molecule that can be elastically bent, elongated and supercoiled negatively or positively. As a matter of fact, in living cells, the molecule keeps on being constrained by thermal agitation and even more dramatically by the constant action of various molecules. For example, the action of polymerases will induce strong supercoiling ahead and behind where it acts [30]. Finally, the pitch and bending of the DNA helix keeps on varying locally, depending in particular on the local base composition [31].

Under these conditions, the constraint on the precise position in the genome of the conserved nucleotides of an interaction site is low. Indeed, when one conserved nucleotide of a given pattern is shifted from one base pair in the genome, chances are high that one of the probable conformations of the DNA molecule will place this nucleotide at the same spatial position compared to when it is not shifted in the genome and with another conformation of the DNA molecule. This is obviously true only if the shifts are not too important. This tends to confirm that class A flexible patterns define protein interaction sites. Indeed, we observed that from one occurrence to the next, the relative position of nucleotides composing them can vary from one to two base pairs. This is small enough so that there exists a likely conformation of the DNA molecule suitable to make it interact with its associated rigid protein. Alternatively, locally constrained DNA segments (for example through preexisting interaction with particular factors) might interact with proteins with flexible segments. Note that the absence of strong constraints on the position of the conserved nucleotides in class A flexible patterns is not easily compatible with other biological functions.

Fifth argument: the presence of optional peripheral repeats of Ts extending the skeleton at its two sides in class A flexible patterns (see Figure 2), can easily be accounted for under this DNA-protein interaction hypothesis. There are at least two ways to interpret the presence of the peripheral repeats. A first idea is to suppose that they could be used by the DNA molecule to stabilize an interacting protein, as they appear on the same side of the DNA molecule as the rest of the conserved nucleotides of the pattern. These peripheral repeats would not be essential in the interaction, which would be possible only when the central part of class A flexible patterns is involved. A second idea is that the peripheral repeats of Ts in class A flexible patterns may help proteins slide along the DNA molecule in order to reach rapidly the central part of the patterns.

Now we may wonder which interacting proteins could be involved. Here is a few requirements that must be fulfilled by proteins to be good candidates according to the features of class A flexible patterns. First requirement: proteins have to be present in large enough amount in cells in order to be good candidates. Indeed, there are many interaction sites defined by the occurrences of class A flexible patterns in genomes. Alternatively, they may be involved in a dynamic process progressively threading the whole DNA molecule through a ratchet-like mechanism (for example forcing DNA segregation into daughter cells). Second requirement: proteins must not play a role exclusively in the transcription process as the pattern occurrences can be found inside coding regions as well as outside. Third requirement: the interaction sites of proteins with the DNA molecule must not be rigidly defined, as the sites we have uncovered in the present study have never been found previously. The fourth requirement that these proteins must fulfill is related to their presence in the organisms of interest. For each candidate protein, we checked whether its distribution in organisms matched the distribution of class A flexible patterns presented in Table 1. Here are some example of plausible candidates: archaeal histones [32,33], histone-like proteins H-NS and IHF [34-40], two topoisomerases (the reverse gyrase and the topoisomerase IIB-VI) [41-44] and the SMC family of proteins [45-49].

Since the patterns are ideally shaped to display specific but labile interaction with proteins, and since they are densely present in genomes with no relationship to the position of genes, we propose that they may be involved in some biological function such as the shaping of the prokaryotic nucleoid or its segregation before cell division.

### Class A flexible patterns could be recognized during homologous recombination

The widespread distribution of flexible patterns of class A along genomes is consistent with selection of the motifs through processes that are fairly ubiquitous and happen sufficiently often in the life of an organism to provide some selective advantage. Until now we have mostly considered structural or regulatory processes involving the DNA molecule as a whole. In the course of evolution the process of recombination plays an essential role as it both permits proof-reading and insertion or deletion of DNA segments. In prokaryotes, recombination involves the formation of long helical filaments of the RecA protein double-stranded DNA [50] and homologs exist in eukaryotes [51]. During the process of recombination, the DNA double helix is distorted, asking for a nucleation process of the first RecA proteins binding, making use of the flexibility of the DNA molecule. The class A flexible patterns, distributed throughout genomes, and insensitive to the origin of the DNA (regions of the genome which are from horizontal gene transfer descent are as likely to harbour the patterns as are the core regions), might play such role. Exchange of base pairs between segments undergoing recombination is essential for recognition of homology, and physical evidence indicates that such an exchange occurs early enough to mediate recognition at A:T base pairs [52]. The conserved skeleton of the class A flexible patterns would provide the required biochemical basis for the process.

## Conclusion

In this article, the source of the ubiquitous bias of period 10–11 bp in genomes has been identified. It is generated by specific and ubiquitous sequences that we named "class A flexible patterns". These patterns are flexible patterns whose main property is to display 10 bp-11 bp periodic repeats of Ts. As the patterns are densely spread in genomes, their occurrences naturally generate the bias.

The patterns account for the second largest bias in the nucleotides distribution of prokaryotic genomes, second to the one generated by the use of genetic code in genes, hence their biological function has to be of an essential nature. We discussed what this function could be and suggested that class A flexible patterns could be defining a new category of protein-DNA interaction sites in genomes.

## Methods

First we introduce the definition of a correlation function which is used throughout this article. Then we explain the theoretical basis of the program we designed to find the sequences responsible for short-range biases, its actual implementation and its controls.

### The correlation function

#### Definition – a genome *G*

A genome *G *of length *L*_*G *_is written  with ∀*i *∈ [1..*L*_*G*_], *x*_*i *_∈ {*A*, *T*, *G*, *C*}. It is taken in the standard 5'-3' orientation.

#### Definition – a sub-genome *S *extracted from a genome *G*

Let  be a genome. A sub-genome *S *of length *L*_*S *_extracted from *G *is a sub-series of *G*. We call *E*_*sg *_(*G*) the set of all the sub-genomes of *G*. Then, for *S *∀ *E*_*sg *_(*G*) composed of *N*_*S *_nucleotides, ∃*σ*: [1..*N*_*S*_] → [1..*L*_*G*_] a strictly increasing function so as .

#### Definition – a pattern *m*

A pattern *m *composed of *N*_*m *_nucleotides and of length *L*_*m *_is written ; *N*_*m *_≥ 1, *p*_1 _= 1,  = *L*_*m *_with *p *a strictly increasing series and ∀*i *∈ [1..*N*_*m*_], *x*_*i *_∈ {*A*, *T*, *G*, *C*}. We call *E*_*m*_(*N*, *L*) the set of patterns composed of exactly *N *nucleotides and with a length shorter or equal to *L*. We call .

#### Definition – an occurrence of a pattern *m *in a genome *G*

Let  be a pattern composed of *N*_*m *_nucleotides and of length *L*_*m*_.

Given the sub-genome  composed of *N*_*s *_= *N*_*m *_nucleotides, *S *is an occurrence of *m *in *G *if and only if ∀*i *∈ [1..*N*_*m*_], *x*_*i *_= *y*_*σ *(*i*) _and *p*_*i *_= *σ*(*i*) - *σ*(1) + *p*_1_. We call *E*_*oc*_(*m*, *G*) the set of the occurrences of *m *in *G*. # *E*_*oc *_(*m*, *G*) is the number of occurrences of *m *in *G *and # *E*_*oc *_(*x*, *G*) is the number of occurrences of the single nucleotide *x *in *G*.

#### Definition – the correlation function *f *(*G*)

Given a genome *G*, an order of correlation *O*_*cor *_and a length for the computation of the correlation *L*_*ana*_, we define the correlation function *f *(*G*) on the space *E*_*m *_(*O*_*cor*_, *L*_*ana*_): for , .

Our practical calculation of correlation functions is performed as follows: the function is represented by an array of size . For each nucleotide of *G*, the array cells of all the patterns composed of *O*_*cor *_nucleotides included in the next *L*_*ana *_bp are increased by one. The number of steps is then proportional to .

The correlation functions of all prokaryotic and lower eukaryotic genomes reveal a strong statistical bias of period 3 bp due to the dense presence of genes in genomes [1]. This bias is of little interest as its source is known. In order to study the other biases in the present work, we always pre-treated the correlation functions so as to hide this trivial bias. This deconvolution step was performed by subtracting the correlation function of a model genome constructed so as to contain only the trivial bias. The concept of model genome has been developed in [53,54]. This is performed here as follows:

#### Definition – the model genome *G*_*mo*_(*G*)

Let us write the genome *G *as a series of dihexanucleotides:  with *H *= (*x*_1_*x*_2_*x*_3_*x*_4_*x*_5_*x*_6_) representing an hexanucleotide.

The model genome *G*_*mo*_(*G*) is a random genome built from *G *by following these probability rules:



#### Definition – the treated correlation function *f*_*t *_(*G*)



The upper line means that *f*_*t *_(*G*) is the average of correlation functions of several model genomes derived from the same genome *G*. The treated correlation function is an average of probabilistic functions. Practically, for genomes long enough, after averaging over a few model genomes (usually three) one gets a function that almost completely lost the effects of biases with very short ranges (inferior to 6 bp) and hence lost the effect of the 3 bp periodic bias due to the presence of the genes, but saved most of the effects of other kind of information included in genomes. In the Background section, on Figure 1, we plotted *f*_*t *_(*G*) restricted on the following set of patterns: (*A*, *A*,1,*l*)_*l*∈[1..100]_.

#### Definition – the complementary sub-genome  of the sub-genome *S*

Given a genome *G *and a sub-genome *S*, we define naturally  as the sub-genome of *G *which includes in the right order all the nucleotides of *G *which are not in *S*.

#### Definition – the model genome *G*_*mo*_(*S*) for a sub-genome *S*

Let  be a genome,  be a sub-genome of *G*,  be its complementary sub-genome and  be a model genome derived from *G*.



#### Definition – the treated correlation function of a sub-genome *f*_*t *_(*S*)

*f*_*t *_(*S*) = *f*_*t *_(*G*_*mo *_(*S*))

#### Notation – a pattern family *M*

A pattern family *M *is a finite set of patterns. It is noted , ∀*i *∈ [1..*N*_*m*_], *m*_*i *_∈ *E*_*m*_.

In the Results section,  and . On Figure 5, 6, we plotted two correlation functions of those two sub-genomes restricted on the following set of patterns: (*A*, *A*,1,*l*)_*l*∈[1..100]_.

### The rationale of the program

Our goal was to determine which sequences of a given genome *G *account for the statistical bias of period 11 bp affecting the distribution of its nucleotides. We designed a program meant to find out which sequences were responsible for all short-range non-trivial biases present in a given genome *G*. Here, "non-trivial" means different from the bias of period 3 bp due to the presence of the genes in genomes. Since the bias of period 11 bp is indeed a short-range bias, the sequences of *G *generating the bias should be included in the sequences determined by the program. Assuming that the majority of significant statistical biases present in a genome *G *can be revealed by the correlation function of *G*, our program does not look directly for the sequences generating the short-range biases but, rather, identifies the sequences generating *f*_*t *_(*G*) for a given *O*_*cor *_and *L*_*ana *_(practically four nucleotides and thirty base-pairs). The treated correlation function of a genome that would be biased only by the genes structure is the null function. Our program stands on the approximated formula (1) that we are introducing now.

#### Definition – a special pattern family for the genome *G*

A pattern family *M *will be called "special pattern family" if (*E*_*oc *_(*m*, *G*))_*m*∈*M *_covers exactly, with no overlapping, the sequences of *G *that generates *f*_*t *_(*G*) for a given *O*_*cor *_and *L*_*ana *_and if the positions of the occurrences of the different patterns of *M *are not correlated. These conditions are written:





We call *E*_*spe *_(*G*) the set of all special pattern families of *G*.

Assuming that such families containing only short enough patterns (shorter than one hundred base-pairs) exist, the aim of our program was to determine one of them.

#### Definition – the simulated genome *G*_*sim *_(*G*, *m*, *β*)

For a given pattern *m*, let *G*_*sim *_(*G*, *m*, *β*) be the simulated genome derived from a genome *G *and constructed by repeatedly overwriting the pattern *m *on the original sequence of *G *(with a frequency *β*). We call *E*_*ocin *_(*m*, *G*_*sim *_(*G*, *m*, *β*)) the set of all the occurrences of *m *artificially introduced in *G*_*sim *_(*G*, *m*, *β*).

**Property – for ***M *∈ *E*_*spe*_(*G*) **and **,



Indeed, we have . As , we have .

Considering the way we derived the simulated genomes, it is obvious that the occurrences of the patterns *m *introduced in *G*_*sim*_(*m*, *G*, *β*) are not correlated to neighboring sequences. We then assume that natural occurrences of *m *in *G *are not too much correlated to neighboring sequences. Hence one gets:



As we introduced the occurrences of the pattern *m *in a non-correlated manner in *G*_*sim *_(*m*, *G*, *β*), it results that .

We have  because many occurrences of the pattern *m *have been introduced in *G*_*sim *_(*m*, *G*, *β*), generating very strong correlations. Hence . As many more occurrences of the pattern *m *were introduced in *G*_*sim *_(*m*, *G*, *β*) than there are naturally in *G*, one has . Finally it comes that .

Hence the treated correlation function of *G *can be approximated by a linear combination of the correlation functions of the simulated genomes associated to the patterns belonging to a special pattern family. This property gave us a theoretical framework to determine such a special pattern family.

#### Definition – a positively free family

Let *E *be a vectorial space and *F *a family of vectors.

Let us define . The family  is positively free in *E *if and only if 

Our idea was to choose a pattern family *M*_*input *_containing as many patterns as possible that is positively free. If there exists one and only one special pattern family *M*_*spe *_included in *M*_*input*_, then there exists a linear decomposition of *f*_*t *_(*G*) on the  with positive coefficients (for any *β *so as ), i.e. . As this decomposition is unique, by calculating the decomposition of *f*_*t *_(*G*) on the , one can determine which patterns belong to *M*_*spe*_. Hence basically our program, for an input of a genome *G *and a pattern family *M*_*input*_, chose a suitable *β*, calculated the  and the unique decomposition with positive coefficients of *f*_*t *_(*G*) on these functions. It gave as an output a pattern family *M*_*output *_which consisted in the patterns of *M*_*input *_for which the treated correlation functions of the associated simulated genomes are involved.

### Practical implementation of the program

First of all, we assumed that, for *O*_*cor *_= 4 and *L*_*ana *_= 30 *bp*, there exist *N *> 0 and *L *> 0 so that there exists one and only one special pattern family included in *E *(*N*, *L*).

Because of computational time limitation, only input pattern families that are not containing too many patterns (less than one thousand patterns) could be tested. To extend the output possibilities of the program, we ran it in a few steps, at the cost of further approximations. First, we entered *M*_0 _= *E*(2,14) ∪ *E*(3,14) as an input family (this family is positively free). As we did not expect any special pattern family to belong to *M*_0_, we did not calculate the decomposition of *f*_*t *_(*G*) on , but rather a "positive projection" of *f*_*t *_(*G*) on .

#### Definition –  the positive projection of  a vectorial space of finite dimension in the non-void family 

Be < > a scalar product in *E *and || || the associated norm. It is possible to prove that  so as , . We call this vector , the positive projection of  in *F*.

We calculated . The coefficients of this positive projection can be assimilated to a frequency of patterns present in *G*, expressed in *bp*^-1^. Then we constructed *M*_1 _the output pattern family with all the patterns of *M*_0 _for which the coefficient of the treated function of the associated simulated genome is large enough. The selectivity of the program is adjustable at this level. Practically, we kept the patterns for which the coefficients are above , with an average approximately , which makes usually approximately twenty patterns. This is a first approximation in our program. As a second step, we used *M*_2 _as an input pattern family. *M*_2 _is containing *M*_1 _plus all the patterns that can be built by extending the patterns of *M*_1 _with one extra nucleotide. The added nucleotide can be placed at any position inside the original patterns or at their sides (as far as 15 bp from the extremities of the original patterns). Again, we calculated a positive projection and got a resulting pattern family *M*_3_.

We repeated this step as long as we got patterns that were strictly included in  (i.e. all the patterns that are composed of up to six nucleotides and span less than 30 bp). We got usually close to one hundred patterns in this pattern family. Let us call *M*_*final *_this resulting pattern family. It is an approximation of *M*_*spe*_. Then, by merging the patterns (composed of six nucleotides) that could be identify as subsets of a same longer pattern (composed of more than six nucleotides), we obtained patterns that belonged to  while becoming closer to *M*_*spe*_. Finally, from the patterns contained in *M*_*final*_, we could define approximately twenty flexible patterns per organisms (see the Results section).

Besides the approximation generated by the division of the program into a few steps, a few more approximations were introduced during that process. First, the calculation of the positive projection was performed approximately so as to save calculation time. Second, the correlation functions were calculated on restricted sets, practically on *E*(4,30), i.e. *O*_*cor *_= 4 and *L*_*ana *_= 30 *bp*. This made the description of patterns approximate since we aimed at determining patterns containing more than four nucleotides. The correlation order should be longer than the maximum number of nucleotides we want to find in patterns, otherwise the program may find patterns which are actually artefacts (a mix of genuine patterns present at distinct locations in the genome).

The program was written in C code. Built and operated in this way, the program was run on a genome of 2 Mbp in 3 weeks with a 1.8 Ghz G5 CPU. The most time-consuming step is the calculation of the correlation functions with *O*_*cor *_= 4 and *L*_*ana *_= 30 *bp*.

### Controls of the program

Different controls were performed to test the selectivity of the program. First, when run on completely random genomes, the coefficients of the first positive projection were below the threshold, so that the resulting pattern family was empty. Second, the program was also tested with artificial genomes built from completely random genomes in which we introduced a given pattern at random locations. The program proved able to extract the pattern back provided that the pattern frequency of introduction was above . Third, the program proved able to identify already known rigid patterns in genomes (see the Results section).

## Authors' contributions

EL designed the algorithm and performed the bulk of the outlined study. AD proposed the rationale for the study and outlined its biological implications. Both authors participated in the writing of this article.
